# 

**Published:** 1912-09

**Authors:** 


### CONTENTS.

ORIGINAL COMMUNICATIONS:—

Radical Surgery in Cases of Cancrum-Oris or "Noma", with Report of a Case of Bi-Lateral Infection Complicating Typhoide Fever with Recovery. By Baxter S. Moore, M. D., of Atlanta, Ga., and Dr. John S. Clifford, of Charlotte, N. C. ........ 287

Intestinal Obstruction, By H. Stokes Monroe, A. B., M.D., Columbus, Ga. ........ 290

The Relation of the Nervous system to Tuberculosis. By J. Cheston King, A. B., M. D., Atlanta, Ga. 297

Fracture of the Patella. By W. L. Cooke, M. D., Columbus, Ga. ........ 302

Water—Some of its Physiological and Therapeutical Uses. By J. H. Neall, M. D., Atlanta, Ga. ........ 305

Intrahepatic Hemorrhage of Traumatic Origin; Operation: Recovery. By Gaston Torrance, M. D., Birmingham, Ala. ........ 312

EDITORIALS ........ 315

NEWS AND NOTES ........ 317

BOOK REVIEWS ........ 342

